# EPR Effect-Based Tumor Targeted Nanomedicine: A Promising Approach for Controlling Cancer

**DOI:** 10.3390/jpm12010095

**Published:** 2022-01-12

**Authors:** Jun Fang

**Affiliations:** Laboratory of Microbiology and Oncology, Faculty of Pharmaceutical Sciences, Sojo University, Kumamoto 860-0082, Japan; fangjun@ph.sojo-u.ac.jp; Tel.: +81-09-326-4137

Cancer remains the major threat to human health in most advanced countries in the world. Among the three major standard cancer treatments, i.e., surgery, radiation therapy, and chemotherapy, to date surgical removal is still the most effective therapeutic; however, cancer patients who could benefit from surgery are limited. For many cancer patients, chemotherapy is the final and important option. Although there is more than 70-year history of chemotherapy, conventional chemotherapy is far from successful. The major problem derives mostly from the lack of tumor selectivity; anticancer drugs are distributed not only in cancer but also in normal tissues. At the same time when they kill cancer cells, they also do harm to normal cells. The non-selective delivery of cytotoxic drugs induces severe adverse side effects that many cancer patients suffer from, which will also limit the usage/dosing for anticancer drugs, resulting in less antitumor effects. Thus, the development of therapeutic strategies with high tumor selectivity is urgently needed, and targeted anticancer therapy has become a focus of cancer research.

In this concern, molecular-target drugs have been extensively developed in the past two decades, which usually focus on essential kinases or receptors highly expressed in tumors. However, with the deepening of research, many limitations and drawbacks of molecular-target drugs have been recognized, and the major concern is the intrinsic heterogeneity of human solid tumors. Thus, anticancer spectra of molecular-target drugs are very narrow, and personalized medicine or precision medicine is necessary to achieve satisfactory effects, which results in enormous expenses of these drugs, including their toxic effects. More recently, immunotherapy has received extensive attention by focusing on immune escape mechanisms; however, similar problems to those of molecular-target drugs exist, which may become a hurdle of this anticancer strategy.

While molecular-target drugs target cancer at the molecular level, at a much earlier period of time, a more general tumor-targeting strategy had been depicted and developed by focusing on unique anatomical and pathophysiological features of solid tumors [1]. Compared to normal blood vessels, tumor blood vessels are very leaky due to the defected architecture of endothelial cells and high vascular permeability due to the highly expressed vascular mediators such as bradykinin (BK), nitric oxide (NO), and vascular endothelial growth factor (VEGF), by which the accumulation of macromolecules (i.e., larger than 40 kDa) selectively into tumor tissues could be achieved with very little distribution in normal tissues [1,2]. This unique phenomenon is coined the enhanced permeability and retention (EPR) effect, and it was first recovered by Matsumura and Maeda in 1986 [1], which is a landmark principle in the development of targeted anticancer drugs.

Based on the concept of the EPR effect, macromolecular anticancer strategy, i.e., nanomedicine, has been developed. Tumor-targeted drug delivery systems using nanoplatforms including liposome, polymeric micelles, polymer conjugate, and nanoparticles have become a promising fusion area for nanotechnology and medicine. In the past two decades, many researchers have been working on EPR effect-based nanomedicine, taking an enormous step forward. In 1980s, the founder of EPR effect, Professor Maeda, developed styrene maleic acid copolymer conjugated neocarzinostatin (SMANCS), which was approved in Japan in 1990s [1]. Recently, more nanomedicines have been used in clinic, for example, Doxil is an FDA approved liposomal drug for the treatment of Kaposi sarcoma and other cancers. Other clinically approved nanomedicine includes liposomal daunorubicin (DaunoXome), liposomal cytarabine (DepoCyt), nonpegylated liposomal doxorubicin (Myocet), pegylated L-asparaginase (Oncaspar), albumin-based paclitaxel nanoparticles Abraxane, and paclitaxel-containing polymeric micelles (Genexol-PM). More nanomedicines are in pre-clinical stage of development [3,4,5,6,7,8,9], all of which show superior tumor selectivity by taking advantage of the EPR effect, resulting in improved antitumor effects with less adverse effects [3,4,5,6,7,8,9].

A critical issue should be addressed is that the EPR effect is the phenomenon of blood vessels, which is largely dependent on tumor blood flow. While most animal solid tumor models that are rich in blood flow exhibit good EPR effect, many clinical cancers, especially advanced late-stage cancers and refractor cancers, are poor in tumor blood flow due to high coagulation activity and thrombi formation, thus showing unsatisfactory EPR effect [1,2,10,11,12,13]. Thus, further augmentation of the EPR effect is of great importance and necessity. In this regard, we should understand that the EPR effect is not a static phenomenon, it is a dynamic event, which could be enhanced by modulating vascular mediators in tumor such as using angiotensin II, NO/nitroglycerin, and angiotensin II, converting enzyme inhibitors and carbon monoxide [1,2,10,11]. A combination of vascular mediators with nanomedicines may become useful strategies for more effective antitumor nanomedicine. Other strategies, for example, by modulating tumor vessels [12] or by targeting tumor stroma and extracellular matrix [13], have also been extensively investigated and proven effective in improving the therapeutic effect of EPR effect-based nanomedicine.

This Special Issue of “EPR effect-based tumor targeted nanomedicine” includes 14 papers from experts working on the EPR effect and nanomedicines of a diverse range of areas. The discoverer of EPR effect, Professor Hiroshi Maeda, and his former students and colleagues summarized the history; principle; the progress and prospects of EPR effect-based tumor targeting strategies; and the application of nanomedicines [1,2,6,8], in which the concept of EPR effect is not only applied to the development of targeted anticancer drugs [3,4,9] but is also applicable for radiation therapy [5], bacterial therapy of cancer [7], and nucleic acid medicine [14]. Moreover, more papers in the Special Issue emphasized the significance of EPR enhancement, discussing the usefulness of potential EPR enhancers [1,2,10,11,12,13], which I believe to be an essential issue for successful anticancer therapy using nanomedicine.

The aim of targeted anticancer research is to develop anticancer drugs with high anticancer effects while showing low side effects, to provide patient friendly service, and to benefit most cancer patients. We believe EPR-based nanomedicine will be a promising paradigm to reach this goal and will be a solution for cancer in the future.

## Data Availability

Not applicable.
